# Current Advancements and Future Road Map to Develop ASSURED Microfluidic Biosensors for Infectious and Non-Infectious Diseases

**DOI:** 10.3390/bios12050357

**Published:** 2022-05-20

**Authors:** Tanu Bhardwaj, Lakshmi Narashimhan Ramana, Tarun Kumar Sharma

**Affiliations:** 1 NCR Biotech Science Cluster, Translational Health Science and Technology Institute, 3rd Milestone, Gurugram Expressway, Faridabad 121001, India; tanu.bhardwaj10@gmail.com; 2 Manipal Institute of Regenerative Medicine, Manipal Academy of Higher Education (MAHE), Bangalore 560065, India; narasimhanbiochem@gmail.com; 3 Department of Medical Biotechnology, Gujarat Biotechnology University, Gujarat International Finance and Tec (GIFT) City, Gandhinagar 382355, India

**Keywords:** diagnostics, biosensors, microfluidics, microfluidics-based biosensors, infectious diseases, non-infectious diseases, aptamers

## Abstract

Better diagnostics are always essential for the treatment and prevention of a disease. Existing technologies for detecting infectious and non-infectious diseases are mostly tedious, expensive, and do not meet the World Health Organization’s (WHO) ASSURED (affordable, sensitive, specific, user-friendly, rapid and robust, equipment-free, and deliverable to end user) criteria. Hence, more accurate, sensitive, and faster diagnostic technologies that meet the ASSURED criteria are highly required for timely and evidenced-based treatment. Presently, the diagnostics industry is finding interest in microfluidics-based biosensors, as this integration comprises all qualities, such as reduction in the size of the equipment, rapid turnaround time, possibility of parallel multiple analysis or multiplexing, etc. Microfluidics deal with the manipulation/analysis of fluid within micrometer-sized channels. Biosensors comprise biomolecules immobilized on a physicochemical transducer for the detection of a specific analyte. In this review article, we provide an outline of the history of microfluidics, current practices in the selection of materials in microfluidics, and how and where microfluidics-based biosensors have been used for the diagnosis of infectious and non-infectious diseases. Our inclination in this review article is toward the employment of microfluidics-based biosensors for the improvement of already existing/traditional methods in order to reduce efforts without compromising the accuracy of the diagnostic test. This article also suggests the possible improvements required in microfluidic chip-based biosensors in order to meet the ASSURED criteria.

## 1. Introduction

**Figure 1:**
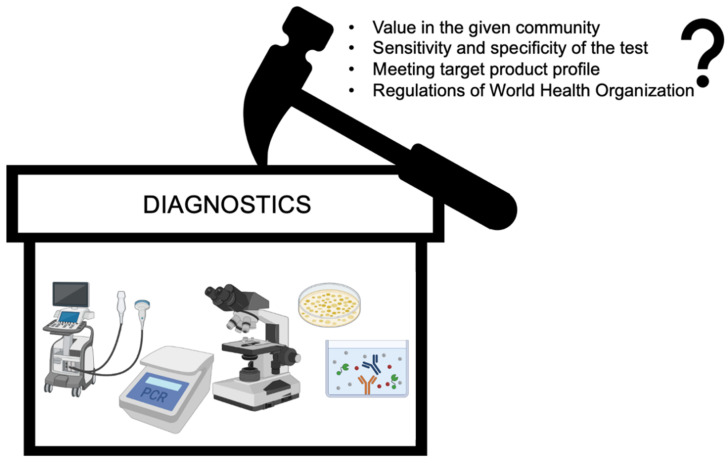
A schematic diagram to demonstrate various restraints in diagnostic techniques. Created using BioRender.com.

**Figure 2:**
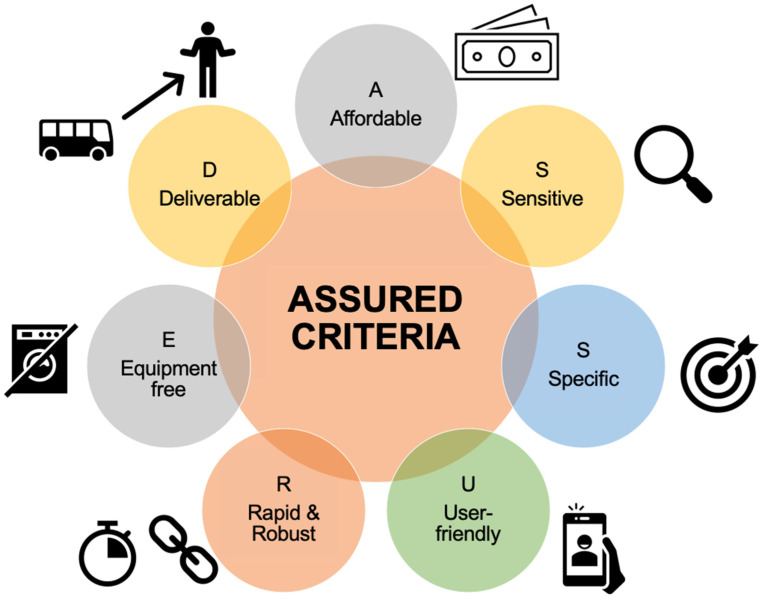
A schematic representation of the WHO’s ASSURED criteria for diagnostics.

Whether a disease is infectious or non-infectious, diagnostics play a vital role in identification of the disease and in seeking medical attention [1]. A medical treatment can not cure a patient or prevent the disease until it is timely and accurately diagnosed. In other terms, it can be said that medicine is blind without diagnostics. If we turn our head to the other side of diagnostics, then the diagnostic techniques are always on a knife edge because of various restraints, such as value in the given community, sensitivity and specificity of the test deployed, meeting the Target Product Profile (TPP) or regulations of the World Health Organization (WHO), and other regulatory agencies (Figure 1) [2,3]. As per the guidelines of the WHO, the diagnostic device/test must fulfill the ASSURED criteria i.e., (i) affordable, (ii) sensitive, (iii) specific, (iv) user-friendly, (v) rapid and robust, (vi) equipment-free, and (vii) deliverable to end-users for the selection of diagnostics for the developing countries (Figure 2) [1,4]. Due to this fact, the field of diagnostics is ever evolving and finding novel technologies for diagnosis of infectious and non-infectious diseases. The characteristics here noted by the WHO for selecting diagnostic devices can be fulfilled by integrating microfluidics and biosensing principles [4,5]. Microfluidics deal with the manipulation/analysis of fluid within micrometer-sized channels and present advantages, such as portability, demand of less sample, option of deployment in remote locations, less power consumption, lower chances of human error, multiple analyte detection, etc. [6]. Biosensors comprise biomolecules immobilized on a physicochemical transducer for the detection of a specific analyte. Biosensors are known for their selectivity and specificity owing to their molecular recognition element component and high analytical sensitivity due to the transducer [7]. The integration of these two different techniques offers all the qualities, such as reduction in size of the equipment, faster turnaround time, possibility of multiplexing, specificity, selectivity, sensitivity, ease of use, etc., which are required for fulfilling the WHO’s ASSURED criteria for selection of diagnostics [8].

Innumerable advancements in technology can be seen in the field of diagnostics for the detection of a variety of infectious diseases. For instance, classical methods, such as microscopy and cell culture, have been replaced with biochemical (immunoassay/colorimetry-based) and advance biotechnology (molecular genotyping, DNA microarray, etc.) methods in which various pathogen related proteins (antigens), toxins, nucleic acids, and antibodies are targeted for diagnosis. Although the classical methods are marked as gold standards and known for cost-effective diagnosis, they have limitations, such as tedious preparations, longer turnaround time, poor sensitivity (in some cases), etc. Conversely, the biochemical and advanced biotechnology methods are faster and more sensitive [2], but their dependence on sophisticated equipment and proprietary reagents limits their application in low and middle income countries. Considering the current situation, the most required characteristics in a diagnostic test/device for infectious disease detection (ASSURED criteria) are rapidity (less than one hour) and easy operation (no dependence on sophisticated equipment) [9]. These two attributes are important for a diagnostic test/biosensor, as they can guide clinicians for a timely and evidence-based treatment and thus effective management of disease. Researchers are already developing handheld or point-of-care testing (POCT) devices for the diagnosis of infectious diseases [10]. POCT can be handed out for faster detection, control, and treatment of infectious diseases, raising the chances of infection-specific treatment [11]. Moreover, POCT can be easily accessed by patients. Microfluidics-based biosensors can be a great choice for the development of such POCT devices. This technology can give rise to faster, simplified, accurate, sensitive, and specific diagnostics [8], which can help in the timely treatment of infectious diseases and would help in stopping the spread of infection.

Conversely, non-infectious diseases, responsible for ~71% of worldwide deaths each year, require diagnosis not only for treatment, but also for prevention and management of the disease. Non-infectious diseases, such as cardiovascular diseases (CVDs), cancer, diabetes, etc., are silent killers. Out of the ASSURED criteria, the most required characteristics for diagnosis of non-infectious diseases are easy operation, rapidity, and affordability. Particularly, the diagnosis of non-infectious diseases relies on biochemical (e.g., biomarkers, glucose, hemoglobin, etc.) or biological (e.g., electrical, neural, muscular, etc.) analysis, which requires laboratory infrastructure and large equipment, which are difficult to setup in low-resource settings. Moreover, a distant laboratory, poor transport of samples, maintenance of quality of samples, and financial burden could be other issues that can hinder the process of diagnosis of non-infectious diseases [12]. Considering this problem, there is a shift in the way we diagnose non-infectious diseases. In the most recent decade, a major focus was placed on point-of-care (POC) diagnosis over centralized laboratory-based investigations, such as in the case of diagnosis and management of diabetes, where we have made substantial progress by creating a handheld glucometer device (POCT device/biosensor) from urine acid hydrolysis and test strips. Moreover, this POCT device has altered the style of diagnosing a patient by increasing the patient’s desire for testing [12]. Next, in the case of CVDs (specially for myocardial infarction (MI)), the benchmark techniques are electrocardiogram (ECG), X-ray, computed tomography (CT), angiography, etc., which are costly and unaffordable in resource-limited developing countries [13]. The current clinical methods for diagnosis of MI (detection of biomarkers such as troponin-T/I, C-reactive protein (CRP), soluble suppression of tumorigenicity 2 (sST2), etc.) have several disadvantages, such as long analysis time, higher expense, and large sample requirement [13,14]. Due to these disadvantages of current techniques, patients suffering from MI experience a delay in initial treatment, which further increases the financial burden and chances of irreversible loss. Currently, microfluidics-based biosensors can be a great choice for handheld or POCT devices for easy-to-use and cost-effective detection, and for management of MI [15]. Similarly, for other non-infectious diseases, microfluidics-based biosensors (POCT) can be highly advantageous from the perspective of early diagnosis and effective management of the disease. 

In this review article, we provide an overview of the history of microfluidics, current practices in the selection of material for microfluidic devices, and how microfluidics has been used for biosensing of infectious and non-infectious diseases. Our inclination in this review article is to show the tendency of microfluidics-based biosensors in the betterment of already existing/traditional methods for detecting infectious and non-infectious diseases. How the existing microfluidic chip biosensors can be improved to fulfil the WHO’s ASSURED criteria is also suggested.

## 2. Brief History of Microfluidics

**Figure 3:**
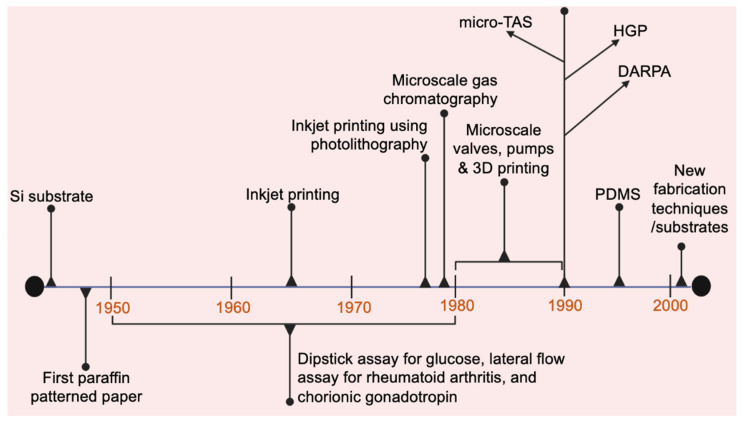
A roadmap highlighting the evolution of microfluidic technologies. Created using BioRender.com. Micro Total Analysis System is abbreviated as Micro TAS, HGP is Human Genome Project and Defence Advanced Projects Research Agency project is abbreviated as DARPA and poly(dimethylsiloxane) as PDMS.

The end of 20th century is known to be a game changer for microfluidics after the invention of transistors (semiconductor industry) in 1947 by William Shockley, Walter Brattain, and John Bardeen [16,17]. A roadmap to present the evolution of microfluidics is shown in Figure 3. The beginning of microfluidics was dominated by silicon (Si) materials [6,18]. The techniques employed for fabrication of silicon-based devices were borrowed from the semiconductor industry [6]. Jay Andrus used the technique of photoengraving (which is presently called photolithography) to fabricate devices demonstrated by Shockley, Brattain, and Bardeen on silicon material. This work was further extended by Jack Kilby, resulting in the development of integrated circuits. The next benchmark product of microfluidics was inkjet printing, as demonstrated by Sweet [18,19,20]. This printer was regarded as the first microfluidic device [16]. Then, Bassous used photolithography on silicon wafers to fabricate an array of inkjet nozzles, which helped in the commercialization of inkjet printers and proved the use of silicon for mass production of microfluidic devices [21]. Another achievement recorded in the history of microfluidics was the microscale gas chromatography system by Terry [16,19,22,23]. Although the terms Lab-on-chip or Micro Total Analysis System (µTAS) were not invented at that time, this system is believed to be one of them [16]. Microscale valves, pumps, and 3D printing were the major accomplishments that were witnessed by microfluidics in the 1980s [16,18]. In the 1990s, the term µTAS was coined by Manz, proposing the idea of performing many functions on a single microfluidic chip [24]. After the birth of µTAS, many projects were bagged by microfluidics, such as the Defence Advanced Projects Research Agency (DARPA), to develop portable devices to detect biological and chemical weapons [16,22]. The other major project was the Human Genome Project (HGP), which mapped entire human genome [16,22,25]. Due to this project, glass entered into the field of microfluidics in order to replace silicon material for biological applications. Alongside this, the tedious technique of gel electrophoresis was replaced by microscale capillary electrophoresis (CE) to ease the sequencing of DNA [16,22,26]. This CE system was demonstrated to be faster and better in resolution than traditional gel electrophoresis. Further, to give rise to a µTAS system for HGP, a microfluidic chip for polymerase chain reaction (PCR) was also fabricated in order to merge it with CE for DNA sequencing application [27]. In the late 1990s, the most popular material in microfluidics was pioneered by Whitesides and his group at Harvard University, called poly(dimethylsiloxane) (PDMS) [6,16]. This material does not force the use of expensive clean room facilities for the fabrication of devices, and the techniques (related to PDMS, such as bonding) are simple [18]. Moreover, use of PDMS was also demonstrated in fabrication of valves and pumps in microfluidic devices [16,22]. In the 20th century, several more advancements were seen in microfluidics, including manufacturing techniques, for example, replica molding, embossing, rapid prototyping, micro injection molding, thermoplastic bonding, plasma processing, etc. [16]. Furthermore, materials that advanced at the same time included paper (different types, such as Whatman paper), SU-8, poly(methyl methacrylate) (PMMA), polystyrene, polycarbonate, etc. [16,18]. If we focus on the parallel growth of other materials, such as paper, then it can be said that the modern-day paper microfluidics evolved from the first paraffin-patterned paper (1949), which guided the transport of fluid due to capillary action (developed by Muller and Clegg) [28]. The next revolutionary paper-based devices, which were developed in the next three decades (1950–1980), were dipstick assay for glucose and lateral flow assay for rheumatoid arthritis and chorionic gonadotropin [29]. The modern generation lateral flow assay device for POC detection comprises stacking of patterned paper, photoresist-patterned lithography, patterned hydrophobic materials, and three-dimensional micropads etc. [30]. The above devices utilize various new technologies in the manipulation of the flow of analytes, immobilization of reagents, and detection methods, such as colorimetric methods for visual readouts, fluorescence readouts using hand-held light sources, and electrochemical assays using digital circuits, etc.

In the current century, the field of microfluidics can be observed as bifurcated into many branches due to continuous efforts of researchers and various evolutions, such as traditional microfluidics, droplet microfluidics, open microfluidics, organ-on-a-chip and paper microfluidics, etc. Microfluidics has reached every industry and is noticeably found in more applications. However, when observing the ratio of commercialized microfluidic devices to research in microfluidics, it is still low due to manufacturing-related problems, such as mass production, cost of development and cumbersome processes, etc. As a result, microfluidics is still searching for the perfect material and manufacturing technique, which can solve these issues. One such branch of microfluidics is paper microfluidics, in which the manufacturing techniques are simple, inexpensive, and faster, and they have the capability of mass production (more chances of commercialization). Moreover, paper microfluidics offer extra benefits from the perspective of a developing country and remote areas [18]. 

This discussion briefed the history of microfluidics, along with how new materials and fabrication techniques entered into the field of microfluidics. The ultimate goal of microfluidics now is to reach every industry and to ease the complicated processes entirely. If we turn our head toward biological applications, then these microfluidic devices have already been used by the integration of manipulation components (valves, mixers, and filters) and analytical/detection techniques (electrophoresis, chromatography, electrochemical, and optical detection) for DNA sequencing and fragment sizing, PCR, amino acid, peptide, and protein analyses, immunoassays, cell sorting and manipulation, and in vitro fertilization [6,31]. Onward in this article, we focus specifically on current practices in the selection of materials for microfluidic devices and diagnosis of infectious and non-infectious diseases using microfluidic devices.

## 3. Current Practices in the Selection of Materials for Microfluidic Devices

In microfluidics, the choice of material decides the fabrication technique. Here, the reason for discussing the selection of material for microfluidic devices is that the material decides the fabrication technique, and both (material and fabrication technique) affect the affordability and commercialization of the microfluidic chip to a major extent, which are the two features of ASSURED criteria for diagnosis. 

**Figure 4:**
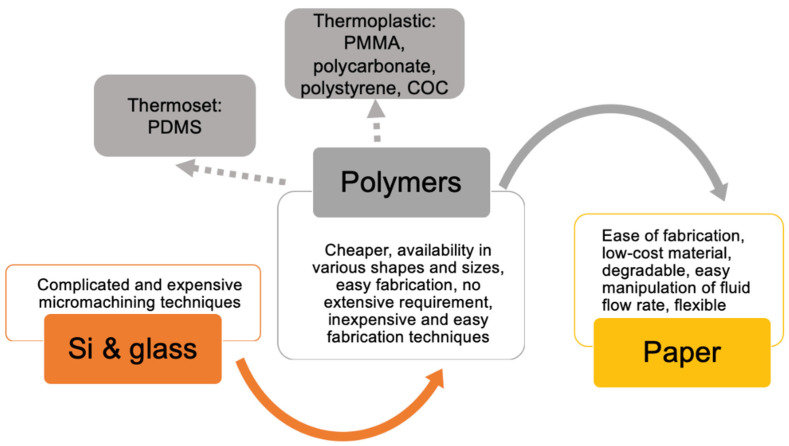
A schematic diagram showing the change in materials with time in microfluidics and their disadvantages/advantages.

A schematic diagram is shown in Figure 4 demonstrating the changes in the materials with time in microfluidic applications. Initial works in microfluidics were majorly demonstrated on materials, such as silicon and glass [32]. These materials required complicated and expensive micromachining techniques, which necessitated extreme training in a clean room and working experience on equipment for wet and dry etching, photolithography, electron beam lithography, etc. [6,33]. Somehow, these fabrication techniques (i.e., materials) hampered the commercialization of microfluidics in the industry. Hence, in order to promote worldwide application or commercialization of microfluidics, those materials entered in industry, which could be cost-effective and require less specialized fabrication techniques [33]. 

Currently, disposable materials, such as polymers, are most likely preferred due to their overwhelming features, such as being cheaper, availability in various shapes and sizes, easy fabrication, no extensive requirements, etc. Microchannels in polymers can be structured by techniques, such as injection molding, hot embossing, replica molding, micromilling, laser ablation, or casting, etc., which are genuinely inexpensive and easy techniques [16,34,35,36,37,38,39,40]. Particularly, due to its soft nature and easy-milling technique, fluoropolymer, such as polytetrafluoroethylene, is recognized as a practical material for constructing microfluidic channels. In addition, this polymer is hydrophobic, which makes it suitable for the flow of fluids within the microchannels. Another category of polymers, i.e., thermoplastic polymers, can be hot-embossed or injection molded for patterning microchannels [36]. 

PMMA is a thermoplastic polymer, which is one of the most commonly investigated materials in microfluidics because it is rigid, inexpensive, optically transparent, suitable for mass production, and compatible with many biomolecular methods. Alongside PMMA, other thermoplastic materials, such as polycarbonate (PC), polystyrene, and Cyclic Olefin Copolymer (COC), are also now considered for designing microfluidic devices, especially for embossed or injection molded parts [16]. These materials have many choices in bonding techniques, such as thermal or glue/solvent-assisted bonding, and are truly beneficial for mass production. 

For rapid prototyping, the usage of thermoset polymer material, such as PDMS, is well documented. For structuring microchannels in PDMS, a master could be made up of silicon or smooth metal for casting [16,41]. The flexibility, low thermal conductivity, hydrophobicity, biocompatibility, low toxicity, low-curing temperature, simple-to-cast ability, and tight sealing with many smooth surfaces, such as glass, hard plastic, photoresists, silicon, silicon nitride, polyethylene, glassy carbon, oxidized polystyrene, fluorocarbons, metals, etc., make PDMS unique and currently most applicable for microchannel fabrication in research laboratories [36,42,43,44,45]. Moreover, the bonding technique used with PDMS is quite simple and reliable. Due to its elasticity, studies have reported the application of PDMS in the fabrication of membranes and valves in microfluidic devices over other polymers [16]. Even more, PDMS has been recorded as the most profoundly used material for microfluidic chip fabrication for different applications, such as biochemical reactions, genome study, chemical reactions, biological detection, etc. [46,47,48,49]. Although PDMS leads as a thermoset polymer, the material shows few limitations, such as swelling upon reaction with many organic solvents (such as oils), absorbing lipophilic molecules, impossible electrode patterning due to its pliability, etc. [42,50,51]. However, it remains unbothered with solvents, such as water, nitromethane, ethylene glycol, acetonitrile, perfluorotributylamine, perfluorodecalin, propylene carbonate, etc. Moreover, its compatibility with organic solvents is usually enhanced by coating it with layers, such as sodium silicate [42]. From the perspective of microfluidic chip fabrication techniques, PDMS again is one of the extensively used materials due to the simplicity in its fabrication method and its ease of bonding with other surfaces [52,53]. The PDMS-based microfluidic chip is assembled by sealing the PDMS microchannels to themselves or to glass, silicon, quartz, etc., via a bonding procedure where glass is preferred due to its comparatively low cost [52]. In addition, glass has the advantage of having patterned electrodes for electrochemical biosensors with great chemical and mechanical stability. The other promising advantage of PDMS/glass chips is rapid prototyping [53]. Hence, the combination of PDMS and glass is recognized to be highly beneficial for the fabrication of microfluidic chip biosensors from the perspective of an efficient product.

The other most preferred material is paper, which is disposable in nature and can give rise to a commercially viable product. Paper microfluidic devices mainly revolutionized in the year 2007 because of ease of fabrication, low-cost materials, degradable materials and manipulation of fluid flow rate, etc. [54]. Paper comprises of cellulose polymers primarily obtained from two raw sources, mainly wood and cotton. Cellulose contains a hydroxy functional group mainly involved in the attraction of water molecules on the surface and thus exhibits capillary action [55]. Moreover, the hydrophilicity of paper can be easily manipulated based on the application, such as for Western blot analysis, in which the hydroxyl groups are esterified in the presence of nitric acid and sulfuric acid to form nitrocellulose filter paper [56]. Generally, for the fabrication of a microfluidic device, Whatman Grade 1 and 4 chromatography paper is used due to the medium-sized porous nature of the material. The physical factors that are to be manipulated for fabrication of a paper microfluidic device are pore size, surface area, capillary action, thickness of paper, tensile strength, electrical properties (such as dielectric constant), etc. [29]. Optimization is mainly based on the analyte properties, reaction volume, detection method, such as either optical or electrochemical methods, etc. In the case of optical-based paper microfluidics, the material or reagents, such as stimuli responsive nanoparticles, aptamers, and antibodies, react with the antigen of the analyte to produce a color at the detection zone, or it uses fluorescence-based detection, etc., which can be visually read out [57,58,59]. Conversely, paper is used in the fabrication of micro-electromechanical system (MEMS)-printed circuit technology due to its inherent and tunable properties, such as flexibility, mechanical strength, and thermal and chemical properties, for electrochemical detection [60]. In flexible paper electronics and wearable devices, fillers, such as gypsum, titanium oxide, clay, graphene, etc., are incorporated into the paper to obtain the desired properties based on the applications [61]. Pencil-drawn components using ionic fluidics are used for sensing temperature, angle goniometer for flexible robots, etc. [62]. Further, paper-based devices are used to measure pH with graphite components and are used for UV sensors with zinc oxide, NO_2_ sensors with silver nanoparticulate, hydrogen sensors on palladium electrodes, etc. All the above materials are coated on paper for sensing [61]. The two main fabrication techniques in paper microfluidics are patterning and customized origami-based methods. The patterning methods are further classified into cutting, printing, deposition, stamping, lithography, and etching [63,64,65,66,67,68,69,70]. Customized devices include modern-day technology involving two-dimensional micropads and three-dimensional micropads (origami-based) [71,72,73,74,75,76].

From the above discussion, it can be concluded that there are numerous materials available for the fabrication of microfluidic chips. A most suited material can be selected in accordance with the requirement of the application. For example, due to the gas permeability of PDMS, it is preferred for biological cell culture applications in comparison to other materials, such as silicon, glass, PMMA, and PC [77]. Conversely, if we look for commercial productions, then PDMS may not be the right choice. For this purpose, thermoplastics can be the ideal material. In addition, thermoplastics require templates in metal or hard material for molding at high temperatures; hence, they are not a great choice for economic research applications (initial mold preparation cost is high). Moreover, from commercialization perspective, mass production and ease in use are mostly preferred attributes for a material. In addition, disposability or single use of the biosensor chip can be a criterion for commercialization. Therefore, plastic or paper could be an ideal choice. For research, PDMS, glass, and silicon are used [77]. Glass and silicon necessitate the use of highly complicated techniques, which are not at all appropriate for scale up and manufacturing perspectives [78]. At the same instance, glass is the ideal material for commercial DNA microarrays due to its high chemical inertness, low-fluorescent background, and transparency [79]. For research applications, PDMS is mostly used to establish proof-of-concept due to its easy availability and access to well-optimized, rapid and straightforward protocols. Its commercialization is hindered mostly due to cost concerns and reusability [78]. Briefly, it can be inferred that there is a huge gap between the selection of materials for laboratory research and commercialization due to various aspects. The right choice of material for one application can be detrimental to use in another. Hence, a thorough navigation of materials is necessary before we proceed toward fabrication of an ASSURED criteria-fulfilling product.

## 4. Microfluidic Chip Biosensors for Diagnosis of Infectious Diseases

**Figure 5:**
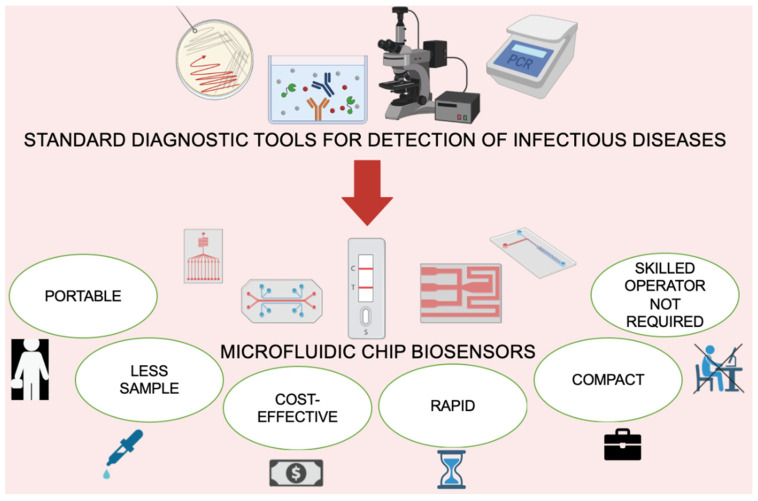
A schematic diagram showing the replacement of conventional techniques for the detection of infectious diseases with new microfluidic chips with advantages, such as portability, less sample requirement, cost-effectiveness, rapidity, small size (compact) and no requirement of a skilled operator. Created using BioRender.com.

Microfluidics-based biosensors can be a great platform to achieve an ASSURED criteria-fulfilling diagnostic product. This integrated technique (microfluidics and biosensors) brings advantages of both the fields together, which can yield a promising device. Microfluidic chip biosensors not only satisfy the necessary requirements for ASSURED criteria, but claim many more advantages, i.e., portability, demand of less samples, option of deployment in remote locations, less power consumption, lower chances of human error, multiple analyte detection, etc., as shown in Figure 5 [80]. Figure 5 also demonstrates the replacement of conventional techniques for the detection of infectious diseases with new microfluidic chip biosensors, which have the mentioned advantages. Moreover, paper-based microfluidic chip biosensors present many more advantages as a diagnostic tool, such as recyclability, disposability, easy transportation, high standard mass production, etc. [58]. The role of microfluidics-based biosensors has already been recorded in different applications, such as environmental analysis, biomedical applications, microscale chemical testing, process analytical techniques, combinatorial synthesis and assays, etc., showing tremendous growth in every possible field [81,82]. On the basis of these facts and features, microfluidic chip biosensors are found to be highly suitable and desirable for diagnostic devices.

In this section, we emphasize on microfluidic chip biosensors for the diagnosis of three infectious diseases, i.e., malaria (caused by a parasite), bacterial sepsis (caused by bacterial pathogens), and acquired immune deficiency syndrome (AIDS) (caused by a virus, HIV). In addition, we describe (i) how already existing techniques do not fulfill the ASSURED criteria of the WHO, (ii) how microfluidic chip biosensors satisfy the ASSURED criteria, and (iii) how further improvements can help microfluidic chip biosensors to meet the ASSURED criteria. 

The contribution of microfluidic chip biosensors in a non-infectious disease, i.e., cardiovascular, is also described in order to show that these microfluidic chip biosensors can be a better diagnostic device for non-infectious diseases. Table 1 summarizes the microfluidic chip biosensors discussed in this and the next section. In addition, target, material for fabrication of the chip, assay principle, detection method, limit of detection (LOD), and the characteristics fulfilled by the chip biosensor out of the ASSURED criteria are also mentioned in Table 1.

### 4.1. Malaria

According to the WHO World Malaria Report, 241 million malaria cases in 2020 were estimated globally [103]. Malaria is a severe health issue worldwide. The gold standard method for detecting malaria is microscopy (thin and thick Giemsa-blood smear) in which a trained technician can detect approximately 1000 parasites per microliter of the sample. Disadvantages of the method, such as the sensitivity of this method, highly rely on the quality of the microscope and skills of the handler, inability to differentiate between different plasmodium strains, transportation (poor roads) of microscope to rural areas for diagnosis, requirement of electricity for microscope in rural areas, etc. These disadvantages have surpassed the advantages of the method, i.e., affordability and rapidity [4,83,104,105,106]. Another diagnostic tool for malaria detection is the rapid diagnostic test (RDTs), in which the monoclonal antibodies used for detection basically search for the malaria antigen in the sample. The best part of this test is that it takes 5–20 min for detection without any requirement of experts for handling and interpretation. Unfortunately, an unfavorable climate can denature the molecules (antibodies) adsorbed for detection on RDTs. Moreover, it is reported that RDTs can detect all plasmodium strains; hence, it is incapable of diagnosing in the case of mixed infections. In addition, the sensitivity of the method is low if the parasitemia level is less than 200 parasites/µL [4,8,83,104,105,106]. In order to improve sensitivity (up to 15–25 parasites/µL) and to detect mixed infections, PCR is used for diagnosis of malaria, but this method suffers from various limitations, such as the method depends on the design of the primers, storage of the chemicals, nucleic acid extraction protocols, contamination, transportation, etc. [4,8]. In all these mentioned methods, no method can reach the early malaria detection limit of 5–20 parasites/µL. It can be observed from this scenario that all these diagnostic methods for the detection of malaria suffer from one or more drawbacks, thus not fulfilling the ASSURED criteria of the WHO. 

To overcome the mentioned issues with existing diagnostics, various measures were taken by researchers, such as introduction of magnetic beads ELISA to increase the sensitivity of the diagnostic method. The antibody-functionalized magnetic beads were used to separate the *Plasmodium falciparum* lactate dehydrogenase (pfLDH) from the parasitized lysed blood, which were then washed, and the activity of the enzyme used was judged using colorimetric enzyme reaction. This increased the sensitivity of the method, as the nonspecific reductase and related species were removed during separation (LOD = 21.1 ± 0.4 parasites/mL) [107]. Similarly, for detection of two malarial biomarkers, pfLDH and histidine-rich protein 2 (HRP2), magnetic microparticles modified with capture antibodies, were used, which sequentially reacted with detection antibodies giving colorimetric changes. The method assured sensitivity, detecting two parasites per microliter [108]. Although ELISA-based methods have achieved reasonable sensitivity for the detection of malarial biomarkers, these methods require large concentrations of antibodies for immobilization, along with reagents (increases expense). Surface functionalization is also complex [83]. Hence, these ELISA-based variant diagnostic methods improved sensitivity but did not meet the other ideas of the ASSURED criteria, such as affordability and equipment-free environment. Viewing all the facts, high sensitivity and affordability along with many more specialties in a malaria detection method have been achieved with the help of microfluidic chip biosensors.

**Figure 6:**
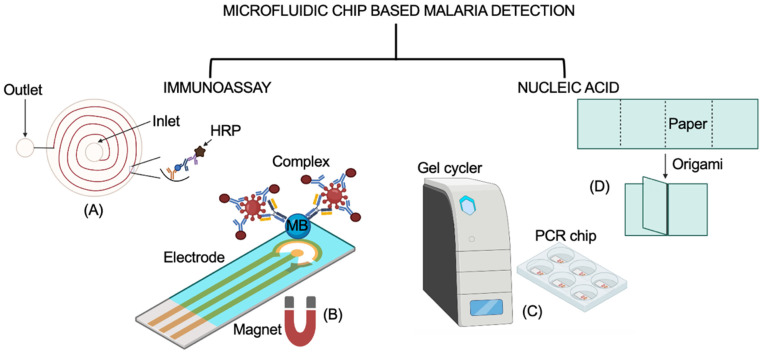
A schematic representation of two types of microfluidic chip biosensors for the detection of malaria disease. (**A**,**B**) are categorized as immunoassay-based microfluidic chip biosensors, while (**C**,**D**) are nucleic acid microfluidic chip biosensors. (**A**) Spiral microfluidic channel for immunoassay. Adapted from ref. [83]. (**B**) Paper-based single-step magneto-immunoassay. Adapted from ref. [84]. (**C**) PCR lab-on-chip with portable gel cycler. Adapted from ref. [8]. (**D**) Three-dimensional micropad origami-folded device for nucleic acid-based assay. Adapted from ref. [85]. The representation of the working principle in this figure explains the mechanism. The original research article representation of these chips may vary from the one shown in the figure. Created using BioRender.com.

Below, we discuss the two types of microfluidic chip biosensors, immunoassay and nucleic acid, which have been used for the detection of malaria disease and have a tendency to fulfill the ASSURED criteria of the WHO. A schematic representation of the working principles of the discussed microfluidic chip biosensors is shown in Figure 6.

#### 4.1.1. Immunoassay-Based Microfluidic Chip Biosensors

Numerous microfluidic chip biosensors have been reported for the detection of malaria. For instance, a microfluidic chip biosensor based on a microplate immunoassay was reported with 1 pg/µL detection of pfLDH (highly suitable from the perspective of the clinical detection range, 3–15 pg/µL) in human serum [83]. In this assay, a spiral microfluidic channel was added instead of a well in a 96-well plate with an inlet, outlet, and spiral channel. Immunoassay was performed within the channel with capture antibody, primary, and secondary-horseradish peroxidase (HRP)-labeled antibody. Quantared™ Enhanced Chemifluorescent HRP Substrate was used for chemiluminescence-based detection of the target. The benefit of this kind of microfluidic well is the reduction of the volume of reagents required for the analysis, using only 5 µL (cost-effective), with an analysis time of 90 min (rapid), user-friendliness, and an increase in the sensitivity of the method. The polystyrene material of the chip further helped in better affordability of the chip. Moreover, a large surface area of the channels in the microfluidic microplate decreased the cases of false-positive results (increased specificity) in comparison to a traditional ELISA plate [83]. Although this chip fulfills many aspects of the ASSURED criteria for diagnostic methods, it can be further improved by using any other HRP substrate that requires simple portable equipment or mobile phone application. For example, instead of using Quantared™ Enhanced Chemifluorescent HRP Substrate for chemiluminescence detection, tetramethylbenzidine (TMB) substrate can be used along with a mobile phone application (Spotxel^®^ Reader) and some modification in the chip design [109]. Not only traditional microfluidics, paper-microfluidics also plays a major role in microfluidics in the demonstration of its application in the detection of malaria. A paper-based POC biosensing device was reported that employed an electrochemical approach to detect malaria disease (pfLDH). The method used a single-step magneto-immunoassay performed with immunomagnetic beads specific to pfLDH, which further reacted to polyclonal antibodies modified with HRP. The complex was added to a single-use microfluidic paper double-sided screen-printed carbon electrode (MP-dsSPCE) below in which a neodymium magnet was placed to immobilize the complex. The method of detection was amperometric. The analysis required 5 min lysis of parasites and 5 min agitation with antibody (specific for pfLDH)-modified magnetic beads before encountering the disposable paper electrode microfluidic device for sample filtration, washing, and detection, which was disadvantage from the perspective of the WHO’s ASSURED criteria. The chip shared many advantages as well, such as detection in less than 20 min, reasonability, and LOD of 300 parasites/µL (close to the WHO’s recommended clinical malaria diagnosis) [84]. 

#### 4.1.2. Nucleic Acid-Based Microfluidic Chip Biosensors

One more microfluidic platform fulfilling almost all the aspects of the ASSURED criteria is a PCR hydrogel plastic (cyclic olefin polymer) chip-based lab-on-chip in which a portable PCR was also designed along with microfluidic plastic chips. The plastic chips contained a polymerized and desiccated hydrogel master mix for PCR amplification, which required direct processing of blood samples (unprocessed sample) without any pre-treatment, overcoming major obstacles in diagnosis. A portable gel cycler was fabricated with a CCD camera for fluorescence detection after amplification. The device identified two major species of *Plasmodium*, *P. falciparum* and *P. vivax* with high sensitivity and specificity. The chips and device were highly reasonable, user-friendly, and the entire system was designed for developing countries. Moreover, the fabricated device required lower power and simple operation from the perspective of fulfilling the WHO’s ASSURED criteria [8]. A paper-based microfluidic chip biosensor using fluorescence assay was developed for the detection of malaria disease. A 3D micropad origami-based folded device was fabricated on which nucleic acid-based assay was performed by DNA extraction from the microbes, loop-mediated isothermal amplification (LAMP), and an array-based fluorescence detection using a portable UV lamp. The efficacy of the kit was assessed with clinical samples and compared with gold standard PCR assays and commercial LAMP assays. The kit was found to be affordable, easy to use (no requirement of skilled operator), sensitive (five parasites per microliter), and able to differentiate between *P. falciparum* and *P. vivax* infection [85].

### 4.2. Sepsis

Another infectious disease is sepsis, which is a life-threatening condition due to an infection. In sepsis, whole body inflammation is observed due to the chemicals (cytokines) secreted by the immune system to fight the infection. It can lead to organ failure, shock, and death; hence, an accurate and rapid diagnosis could help in treatment of this medical condition [4]. The major reasons for sepsis infections are bacterial, viral and fungal infections. In the current review, we are focusing only on bacterial infection. The gold standard method for detection of bacteria in the case of sepsis is bacterial culture, but this method takes several hours (24–36 h) to provide sample-to-answer and requires higher sample volume as well. The other test for detection of bacterial sepsis is PCR, and this method has high sensitivity and specificity as well, but is not suitable for POCT [86,110,111]. In this case as well, the diagnosis methods for bacterial sepsis do not meet the ASSURED criteria of WHO, and microfluidic techniques can be ideal for the same [4,86]. 

**Figure 7:**
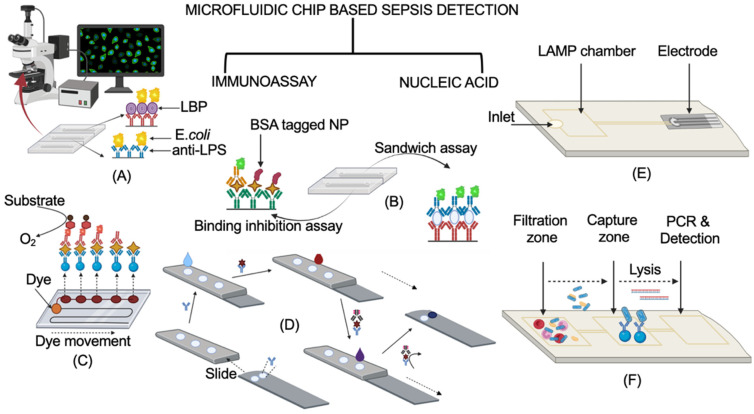
A schematic representation of two types of microfluidic chip biosensors for the detection of sepsis. (**A**–**D**) are categorized as immunoassay-based microfluidic chip biosensors, while (**E**) and (**F**) are nucleic-acid microfluidic chip biosensors. (**A**) *E.coli*-detecting ELISA-based microfluidic chip biosensor. Adapted from ref. [86]. (**B**) Sepsis biomarkers detecting ELISA-based microfluidic chip biosensor. Adapted from ref. [87]. (**C**) ELISA-based POC for sepsis biomarkers. Adapted from ref. [88]. (**D**) Sliding strip 3D-micropad immunoassay-based microfluidic chip biosensor. Adapted from ref. [89]. (**E**) LAMP and hybridization-based electrochemical microfluidic chip biosensor. Adapted from ref. [90]. (**F**) Lab-on-chip for sepsis detection. Adapted from ref. [91]. The representation of the working principle in this figure is to explain the mechanism. The original research article representation of these chips may vary from the one shown in the figure. Created using BioRender.com.

Below, we are discussing two types of microfluidic chip biosensors, immunoassay and nucleic acid, which have been used for the detection of sepsis and have the capability to reach ASSURED criteria of the WHO. A schematic representation showing a working principle of the discussed microfluidic chip biosensors is shown in Figure 7.

#### 4.2.1. Immunoassay-Based Microfluidic Chip Biosensors

Many microfluidic chip biosensors have been reported for the detection of sepsis-causing bacterial pathogens. In one of the microfluidic chip biosensors, green fluorescent protein (GFP)-expressing *Escherichia coli* (*E. coli*) was detected in the blood for diagnosis of sepsis. In the chip, two strategies were used for the detection of *E. coli.* In the first one, *E.coli* was captured by the lipopolysaccharide binding protein (LBP), which was immobilized using anti-LBP antibody on a PMMA/glass microfluidic chip. In the other one, the anti-LPS antibody was immobilized on the chip. For detection, fluorescence microscopy was used. The chip was found to have an LOD of 50 CFUs/mL in blood and had the tendency to detect other sepsis-causing pathogens, such as *Klebsiella*, *Pseudomonas*, *Enterobacter*, *Staphylococcus*, *Streptococcus*, and *Enterococcus* species [86]. The device can be made robust by replacing antibodies with aptamers [112]. In addition, the chip was user-friendly, but required improvement in detection setup in terms of cost-effectiveness and simplicity. 

Biomarkers secreted in the blood in sepsis condition were also targeted by various research groups, for example, a PMMA-based POCT device was fabricated to detect C-reactive protein (CRP) and neopterin (NP). Microchannels for the immunoassay of CRP and NP were designed on the chip and were integrated with a fluorescence detection unit. Sandwich assay was used for CRP (monoclonal antibody and fluorophore-labeled detection antibody) and binding inhibition assay for NP (monoclonal antibody, NP labeled with BSA, and fluorophore labeled detection antibody). The LOD of CRP and NP achieved was 10 and 2.1 μg/L with only 20 min (rapid) of assay time [87]. The optical detection unit was simple, user-friendly, and portable (highly applicable for ASSURED criteria and POCT application). A fully integrated, equipment-free, inexpensive, and portable ELISA-based POC with distance readout was fabricated, which was demonstrated to be applicable for the detection of CRP and other types of biomarkers. In the microfluidic chip biosensor, using a permanent magnet, the capturing antibodies and modified magnetic beads mixed with the sample (antigen) were passed through different chambers containing specific solutions to form bead–capturing antibody–antigen–detecting antibody- streptavidin-biotinylated platinum nanoparticles (PtNPs). In addition to the substrate H_2_O_2_, PtNPs digested the substrate to oxygen, which shifted the position of a prefilled dye displaying a distance signal for the measurement. The chip was found to finish the process within 2 h [88]. Moreover, the device was found to be specific, accurate, and precise along with capability of visual analysis, making the whole system highly appropriate for the ASSURED criteria regarding the diagnosis of sepsis. A sliding strip 3D μPAD-based colorimetric assay using antibody-based ELISA was developed for the detection of CRP to diagnose neonatal sepsis. The antibody used for detection was conjugated with alkaline phosphatase (ALP) using 5-Bromo-4-chloro-3-indolyl phosphate (BCIP), which further reacted with Nitro Blue Tetrazolium (NBT) as a substrate for naked eye visualization, displaying colors ranging from purple to blue. Although the test’s accuracy was not remarkable, the design and principle of the assay were found to be inexpensive, simple to fabricate, portable, easy (no further addition of solutions was required on the chip, except for water and sample), and highly compatible for resource-limited areas [89]. Gold nanoparticles (AuNPs) conjugated with antibody-targeting IL6 were used on paper for the detection of sepsis based on the density of AuNPs binding on the paper immobilized with antibody capturing IL6. The signals were read using a smart phone densitometry app. The method was rapid, highly sensitive, and portable, and required unprocessed blood samples for detection and to meet all the major criteria of the WHO for diagnosis [113].

#### 4.2.2. Nucleic Acid-Based Microfluidic Chip Biosensors

For other sepsis-causing pathogens, such as *Salmonella enterica (S. enterica)*, a microfluidic chip biosensor was fabricated in which a LAMP chamber was designed along with gold-plated electrodes for electrochemical detection [90]. Sequence specific electrochemical DNA probes for two subspecies of *S. enterica*, i.e., *Typhimurium* and *Choleraesuis* were present in the electrochemical detection chamber, enabling detection of and discrimination between the two subspecies. On arrival of the target sequences, methylene blue (MB) attached to the probes displaced from the electrode, leading to a decrease in the current. The direct undiluted whole blood (user-friendly) was used for analysis on the chip. Moreover, the analysis was rapid and was able to detect these two strains at clinically relevant levels (<1000 CFU/mL). In addition, there is a wider scope to further improve the cost of the device using large-scale production. Unfortunately, one limitation of the chip was its small LAMP chamber, which affected its LOD, but it can be modified to increase the sensitivity of the chip. The most recent integrated microfluidic chip for the detection of sepsis-causing pathogens, such as *E. coli*, *Klebsiella pneumoniae*, *Pseudomonas aeruginosa*, *Staphylococcus epidermidis*, and *Staphylococcus saprophyticus*, contains a membrane-based filtration module to remove WBCs and RBCs, a bacteria-capturing module, and a PCR zone for bacteria detection (fluorescence). The chip was able to detect the pathogen within 4 h, with a minimum limit of five CFU per milliliter [91]. The chip was cost-effective, as the materials, such as PMMA, glass, and PDMS, were used for fabrication of the chip. An additional benefit of the chip was that this chip was user-friendly—an example of a lab-on-a-chip. Furthermore, the chip demonstrated better LOD over on-bench PCR due to the removal of interfering substances of blood prior to PCR. However, the chip could be improved in terms of rapidity to meet the ASSURED criteria of the WHO for diagnosis.

### 4.3. AIDS

**Figure 8:**
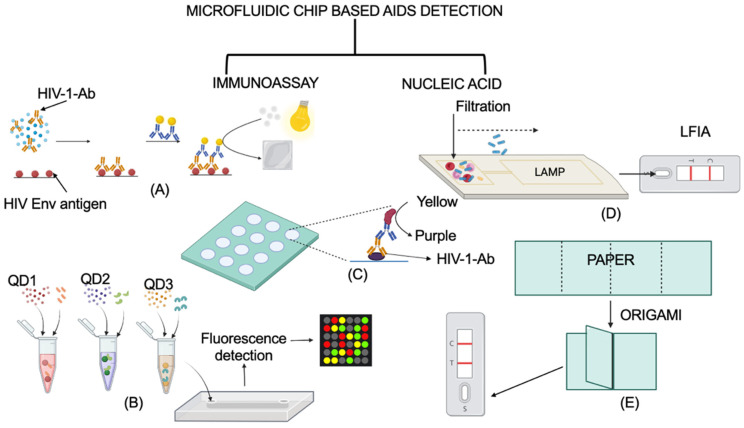
A schematic representation of two types of microfluidic chip biosensors for the detection of AIDS. (**A**–**C**) are categorized as immunoassay-based microfluidic chip biosensors, while (**D**,**E**) are nucleic-acid microfluidic chip biosensors. (**A**) POCKET immunoassay. Adapted from ref. [92]. (**B**) QD-based immunoassay. Adapted from ref. [93]. (**C**) Paper-based ELISA. Adapted from ref. [94]. (**D**) LAMP and LFIA-based sensing. Adapted from ref. [95]. (**E**) Origami-based isothermal, enzymatic amplification of HIV DNA and LFIA sensing. Adapted from ref. [96]. The representation of a working principle in this figure is to explain the mechanism. The original research article representation of these chips may vary from the one shown in the figure. Created using BioRender.com.

Not only for malaria and sepsis, microfluidic chips have been fabricated for other infectious diseases, including AIDS (HIV). Below, we discuss the two types of microfluidic chip biosensors, immunoassay and nucleic acid, that have been used for the detection of AIDS. A schematic representation of a working principle of discussed microfluidic chip biosensors is shown in Figure 8.

#### 4.3.1. Immunoassay-Based Microfluidic Chip Biosensors

For the diagnosis of HIV, microfluidic chip biosensors for the detection of the p24 protein (HIV marker) using on-chip ELISA were fabricated [114]. To distinguish different HIV subtypes, an innovative portable and low-cost POCKET immunoassay was described for the detection of anti-HIV1-antibodies in which gold colloid-labeled antibodies were used. HIV Env antigen was immobilized on the PDMS chip, which reacted with target antibodies on addition of the sample. Then, gold colloid-labeled antibodies formed a complex with the target antibodies, which further reduced the silver ions present in the solution to a silver film on the chip, resulting in a change in opacity of the film. An optical detection setup was employed for the analysis. The opacity of the film clearly defined the concentration of the target antibodies. Moreover, the system had no photobleaching effect and could stay stable for months. The analysis required minimal equipment and skill for operation; hence, it can be a successful diagnostic product in the future after improvements to fulfill the ASSURED criteria [92]. Furthermore, to detect co-infections in HIV patients, multiplexed microfluidic chip biosensors were fabricated [115]. Hepatitis B and C are more prevalent in HIV patients; hence, a chip biosensor was designed that was able to diagnose hepatitis B surface antigen (HBsAg), HCV non-structural protein 4 (NSP4), and HIV glycoprotein 41 (gp41). First, three different types of quantum dots were off-chip adhered to three different targets. Then, the complexes were passed through the PDMS chip equipped with a fluorescence detection setup. All the quantum dots had different emission peaks, which became a signature for the target analyte and helped in quantification. The method was found to be 50 times better in sensitivity than FDA-approved diagnostic methods [93]. Cheng et.al. demonstrated a paper-based ELISA chip biosensor, which contained 96 microzone with a hydrophobic barrier and hydrophilic paper. HIV-1 antigen was immobilized in the microzone, and the sample was added to the hydrophilic test region. Human anti-HIV-1 antibodies attached with the antigen, followed by the addition of secondary antibodies conjugated with alkaline phosphatase. The detection of anti-HIV-1 antibodies was based on the BCIP/NBT substrate showing a blue color in the presence of HIV virions. Although the paper-based ELISA had a lower sensitivity than the 96-well plate ELISA, the method was rapid and required a small sample/reagent volume (1–10 μL). This paper-based ELISA was compatible for POC in clinical analysis only and can further be improved in terms of sensitivity [94]. A highly compatible device for POC testing of HIV (fulfilling ASSURED criteria) is 3D origami-based micropads, fabricated for the P24 antigen using ELISA. The device offers electricity- and instrument-free detection, making it simple and easy to operate by untrained end users. The device did not require any excitation source/detection device, as output was a simple colorimetric readout that could be seen visually. In addition, an option of semi-quantification by comparison of color change with a reference color chart was also incorporated [116]. 

#### 4.3.2. Nucleic Acid-Based Microfluidic Chip Biosensors

A POCT Microfluidic Rapid and Autonomous Analysis Device (microRAAD) for HIV based on RNA detection in whole blood was fabricated, which enclosed reagents, fluidic control, and temperature control. Different membranes were used for blood separation and amplification. The RT-LAMP was visualized using Lateral Flow ImmunoAssay (LFIA), which can detect 3 × 10^5^ HIV-1 viral particles, or 2.3 × 10^7^ virus copies per milliliter of whole blood. The test required 90 min to complete the analysis. From the perspective of POC devices, the device was a simple, sensitive, automated, inexpensive, portable platform that enabled automated detection of HIV and other pathogens at the point of care. The user had to perform four simple steps to start the test and could connect the device to a cell phone/computer for temperature heating. However, the shelf-life of the device was less due to storage or reagents within the device; hence, this might be a difficulty regarding the implementation of the device in at-home POC devices. Furthermore, the device fulfills almost all the criteria required for the WHO’s ASSURED criteria for diagnosis [95]. A paper and plastic hybrid microfluidic chip biosensing device capable of performing isothermal, enzymatic amplification of HIV DNA was fabricated for a proof-of-concept study. The method required DNA extraction from dried blood spots and further used an HIV-detecting LFIA with a control and test line. The proof-of-concept study was successful in terms of LOD (10 copies of HIV DNA could be detected), portability, short detection time (15 min), easy operation, and compatibility with LFIA. However, the method required micropipettes and a heater for operation; hence, it can be suggested that the device can be used as POC (ASSURED criteria) in clinical analysis only [96]. 

## 5. Microfluidic Chips for Diagnosis of Non-Infectious Diseases

Microfluidic chips have contributed not only to the diagnosis of infectious diseases, but also to non-infectious diseases. Microfluidic chips have been fabricated to detect the top non-infectious diseases, such as cardiovascular (CVDs), cancer, and diabetes. Here, in this review, we focus on CVDs to describe the contribution of microfluidic chip biosensors in the detection of non-infectious diseases. 

CVDs are responsible for 17.9 million deaths each year globally [14]. Diagnosis and evaluation of the disease play an extremely important part for CVDs, as these diseases progress rapidly and happen abruptly [14]. Biomarkers, such as heart-type fatty acid binding protein (h-FABP), myoglobin (myo), cardiac troponin I/T (cTnI/cTnT), B-type natriuretic peptide (BNP), N-terminal prohormone B-type natriuretic peptide (NT-proBNP), CRP, creatine kinase-MB (CK-MB), and fibrinogen, are known to be the best diagnostic indicators of the progress and treatment of CVDs [13,14]. The current clinical methods for the diagnosis of CVDs have several disadvantages, such as long analysis time, high expense, and large sample requirement. Moreover, the other instrumentation-based methods, such as X-ray, CT, angiography, ECG, etc., are costly and unaffordable in resource-limited developing countries [13]. Microfluidic chip biosensors are well suited from the perspective of targeting these three disadvantages and can meet the requirements of better diagnostic tools for CVDs [14]. In addition, these chips have an advantage in developing countries with limited health resources due to their inherent features of user-friendliness and cost-effectiveness [13]. 

**Figure 9:**
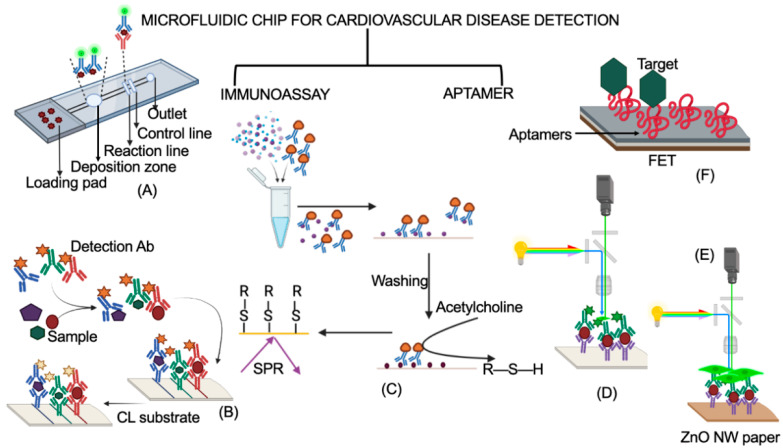
A schematic representation of two types of microfluidic chip biosensors for the detection of cardiovascular diseases. (**A**–**E**) are categorized as immunoassay-based microfluidic chip biosensors, while (**F**) is a nucleic-acid microfluidic chip biosensor. (**A**) LFIA-mimicking silicon-based microfluidic chip biosensor. Adapted from ref. [97]. (**B**) Miniaturized immunosensing microfluidic chip biosensor. Adapted from ref. [98]. (**C**) Snail-shaped chemiluminescence based microfluidic chip biosensor. Adapted from ref. [99]. (**D**) Sandwich immunoassay-based microfluidic chip biosensor. Adapted from ref. [100]. (**E**) Fluorogenic immunodevice. Adapted from ref. [101]. (**F**) Aptamer and FET-array-based microfluidic chip biosensor. Adapted from ref. [102]. The representation of the working principle in this figure is to explain the mechanism. The original research article representation of these chips may vary from the one shown in the figure. Created using BioRender.com.

Below, we discuss two types of microfluidic chip biosensors, immunoassay and nucleic acid, which have been used for the detection of CVDs and have a tendency to meet the ASSURED criteria of the WHO. A schematic representation of a working principle of the discussed microfluidic chip biosensors is shown in Figure 9.

### 5.1. Immunoassay-Based Microfluidic Chip Biosensors

Many LFIA-based methods are commercially available for CVD tests, but it has been reported that they have poor LOD and detection range (e.g., cTnI, LOD in ng/L) [13]. To solve this issue, various measures have been taken, such as magnetic beads labeled with orientation-controlled antibodies for the detection of cTnI, which have been used in LFIA [117]. This modification has helped in increasing the detection sensitivity of LFIA to be as low as 10 ng/mL. Similarly, one more study was conducted for proof-of-concept, which can further be used to enhance sensitivity of LFIA by using dual AuNPs. The LOD for cTnI and myoglobin was found to be 1 ng/L and 1 ng/mL, respectively [118]. 

A microfluidic chip biosensor for the detection of CRP was fabricated, which mimicked LFIA and improved its sensitivity. The chip was designed on a 4-inch silicon wafer that included a sample collector, delay valves, flow resistor, deposition zone, reaction chamber, capillary pump, and vents. The deposition zone was used for storing fluorophore-labeled anti-CRP-C6 detection antibodies. The reaction chamber consisted of planar PDMS lines with anti-CRP-C2 capturing antibody (test lines) and CRP (control lines). The lines were observed using a fluorescence microscope in this study, and the method had a detection range from 1 μg/L to 1 mg/L for CRP. As this method uses expensive equipment for detection, this necessarily requires improvisation in terms of a detection unit [97]. In a further study, the chip was improved for multi-parametric immunoassays [119]. A miniaturized immunosensing microfluidic chip biosensor (glass and PDMS material) using a portable surface plasmon resonance (SPR) sensor was reported to determine BNP for the detection of CVD [98]. The BNP sample was mixed for a few seconds with acetylcholine esterase-labeled antibody (conjugate) in a microvial and then passed into the chip using a syringe pump. Only an unbound conjugate was trapped within the channel, which was already immobilized with BNP. Then, acetylcholine was introduced into the chip, which gave rise to a thiol compound, resulting in the accumulation of the compound on a gold thin film downstream in the channel. This shifted the SPR angle in accordance with the concentration of BNP in the sample. The method was highly sensitive (LOD: 15 fg) and simple (better than multistep assays), but the assay time needs to be improved to meet the ASSURED criteria for POCT application. Further automation is necessary in this system for user-friendly diagnostic application. In addition, BNP-specific aptamers can be used instead of antibodies to increase the robustness of the device [112]. Moreover, real-sample analysis is required to validate the clinical utility of this proof-of-concept. A snail-shaped microfluidic chip biosensor using chemiluminescence (CL) detection was fabricated for the diagnosis of CK-MB, cTnI, and myoglobin [99]. The chip was made up of three layers, in which a silicon film reaction layer was sandwiched between two layers of PDMS (microchannel and base layer). The silicon reaction layer was coated with detection antibodies. HRP-conjugated detection antibodies for each target reacted with the sample and were passed through the chip. The complex of the target and HRP-labeled detection antibodies attached to the immobilized primary antibody for the target. After washing, a chemiluminescent substrate provided the signal for the quantification of targets. In the study, the performance of the chip (CL-based) was found to be much better than other methods, such as colorimetric, immunofluorescence, and surface-enhanced Raman scattering (SERS) in terms of LOD, detection time, sensitivity, portability, and multiple biomarker detection. However, the chip required some pre-processing of the sample. Hence, the chip can be used for clinical POC diagnosis application only, where advanced technologies are not available in the laboratory. In addition, the antibodies could be replaced with robust biomolecules, such as aptamers, to increase the shelf-life of the chip and room temperature storage [112]. A cost-effective, portable, and sensitive biosensing platform was developed on a polymer material, PMMA, using a simple CO_2_ laser-engraving technique for the detection of cTnI. The chip contained capillaries and lenses engraved in the polymer. A portable, low-power, fluorescence excitation and detection device was designed to use in conjunction with the cartridge style chip. In the biosensing of cTnI, a sandwich immunoassay was employed with capture- and fluorophore-tagged detection antibodies. The chip was found to be sensitive for clinical analysis, with a LOD of 24 pg/mL for cTnI and an assay time of 7–9 min [100]. This microfluidic chip biosensor was a full package, having all the necessary features required for a POCT device (ASSURED criteria). Guo et al. reported a fluorogenic immunodevice for the simultaneous detection of multiple cardiac biomarkers, such as h-FABP, cTnI, and myoglobin. The antibodies specific for the biomarkers were immobilized on the paper with zinc oxide nanowires for enhanced sensitivity (5× in comparison to pristine paper) and then incubated with the sample. Further, FITC-labeled detection antibodies for each target were added onto the paper, and fluorescence intensity was measured for quantification. The device showed high selectivity and sensitivity for the biomarkers. Additionally, the assay was simple, inexpensive, rapid (approximately 5 min), quantitative, robust, and portable (required a handheld mobile and UV lamp for detection). Moreover, the device was found to be compatible for POC testing in clinical applications [101]. 

### 5.2. Aptamer-Based Microfluidic Chip Biosensors

For detecting multiple CVD biomarkers, such as CRP, NT-proBNP, cTnI, and fibrinogen, a microfluidic chip biosensor with FET sensor arrays and aptamers as capture probes was fabricated [102]. The chip was composed of PDMS with a separate detection chamber for each biomarker, and the detection was performed by a AlGaN/GaN-based electrical double layer (EDL)-gated high electron mobility transistor (HEMT) sensor. This chip is highly compatible for diagnostics, as this fulfills almost all the perspectives of the ASSURED criteria. The chip was capable of working with unprocessed clinical samples, and the entire system was portable and automated, minimizing human interference (user-friendly). Moreover, the sample volume for the analysis was low (~4 µL), with a detection time of 5 min (rapid). Further, the whole system can be expanded to mass production.

## 6. Future Roadmap

In the future, according to the market analysis of microfluidics, the global market value of this field is expected to reach USD 9538 million by 2026 from USD 4632 million in 2020, with a compound annual growth rate (CAGR) of approximately 12.95% for the period of 2021–2026 [120]. In the key market trends, North America dominates the market of microfluidics and is forecasted to do the same in the period of 2021–2026. Additionally, the United States is predicted to demonstrate a healthy growth in the microfluidics market [120]. Companies, such as Abbott Laboratories, Danaher Corporation, Beckman Coulter Inc., Siemens AG, and Abaxis, etc., are running the POCT market [121]. The number of companies is expected to increase in the near future. A major propelling area of growth in microfluidics is diagnostics due to the continuous rising demand of POCs in the healthcare industry [122]. Although researchers and industries are working hard to bring microfluidics to the market, there are still only few microfluidic chips that nearly fulfill the ASSURED criteria and are in the market. Customer acceptance, complex and tedious regulatory approval protocols, and market adoption hinder the commercialization of microfluidic technology in the market [123]. Hence, this needs to be tackled to improve this scenario in the future. 

It has already been reviewed in the article regarding how implementation of microfluidics has changed the diagnosis of diseases. The remarkable features of microfluidic biosensors and their capability to easily integrate with other technologies, such as artificial intelligence (AI), synthetic/molecular biology, machine learning (ML), etc., have caliber to provide next-generation diagnostic solutions for infectious and non-infectious diseases [124]. Ideally, for next-generation POC diagnostic devices, the new criteria to fulfill will be REASSURED criteria, where R and E stand for real-time connectivity and ease of sample collection. In that case, integration of microfluidics with smartphones could be the game changer [121]. Such devices would help in sending data, and this would in turn help in the efficient management of diseases. Another approach could be in the form of wearable chips that can be used as tattoos, patches, bands, watches, contact lenses, etc., and can be integrated with smartphones for data collection [121]. These devices are especially crucial for the part of the population suffering from illnesses and requiring timely monitoring. In this context, internet-of-things (IoMT), fifth-generation (5G) communication network, ML, and AI can play a crucial role in the creation of such intelligent, wearable, and automated systems [125,126]. The other feature of REASSURED criteria is ease of sample collection. For this, various other body fluids, such as tears, urine, blood, sweat, and saliva, based POCT devices are going to increase in use, as this option provides convenience to the patient [121]. 

Additionally, nanomaterial (like graphene) and 2D materials (MXenes and borophene) can be the role model for the development of fifth-generation POCT sensors, covering aspects, such as portability, compactness, wearability, smartness, intelligence, multifunctionality, point-of-action, remote access, self-power, AI, ML, cloud computing, 5G technologies, etc. [125,127,128]. 

Alongside, accuracy, sensitivity and specificity and benchmarking against the existing gold standard for such POCT devices could be a roadblocking issue in commercialization; hence, these attributes need timely consideration. In addition to this, while designing a microfluidic biosensor device/chip, disease or problem-specific TPPs should be considered since beginning. This would certainly speed up the translation of research to a commercially viable product [3].

## 7. Conclusions

In the last few decades, microfluidic chip-based POCT devices are observed to show fascinating innovation. The POCT devices have improved disease diagnosis and treatment as well. Low cost, rapid, and robust microfluidic chips are achievable now, but the accuracy, detection unit setup, sensitivity and performance match with the current gold standard are the major issues that need to be resolved as a priority. Still, the market is in search of diagnostic devices that can fulfill the ASSURED criteria and benefit the healthcare industry/patients. This search shows that a perfect device or technology is still missing in the market and is yet to come. Bridging of this existing gap needs sincere and collective efforts from the biosensor community. We strongly believe that in a decade or so, many such technologies will be developed that would fulfill the ASSURED criteria and would provide an accurate diagnostic picture. Thus, it will be interesting to keenly watch how innovations in the field of microfluidic biosensors will create a paradigm shift in the area of in vitro diagnostics and biosensing.

## Figures and Tables

**Table 1:** Summary of the microfluidic chip biosensors for the detection of malaria, sepsis, AIDS, and cardiovascular diseases.

Target	Material	Principle	Detection	LOD	ASSURED Criteria (Fulfilled or Not)	Ref.
**Malaria**						
**pfLDH**	Polystyrene	Immunoassay	Chemiluminiscence	1 pg/µL	Affordable, rapid, user-friendly, sensitivity	[83]
**pfLDH**	Paper	Immunoassay	Electrochemical (amperometric)	300 parasites/µL	Affordable, rapid, sensitivity	[84]
***P. falciparum* and *P. vivax***	Plastic (cyclic olefin polymer)	Nucleic acid	Fluorescence	2 parasites/μL	Affordable, user-friendly, portable	[8]
***P. falciparum* and *P. vivax***	Paper	Nucleic acid	Fluorescence	5 parasites/µL	Affordable, user-friendly, sensitivity	[85]
**Sepsis**						
***E.coli***	PMMA/glass	Immunoassay	Fluorescence imaging	50 CFUs/mL	Low fabrication cost, user-friendly	[86]
**CRP and NP**	PMMA	Immunoassay	Fluorescence	CRP:10 μg/L NP:2.1 μg/L	Rapid, simple, user-friendly, portable	[87]
**CRP**	PMMA	Immunoassay	Colorimetric (visual analysis)	0.01 μg/mL	Affordable, portable, equipment-free	[88]
**CRP**	Paper	Immunoassay	Colorimetric	40 ng/mL	Affordable, portable, equipment-free, user-friendly	[89]
***S.enterica***	PDMS and glass	Nucleic acid (LAMP)	Amperometric	<1000 CFU/mL	User-friendly, affordable	[90]
***E. coli*, *K. pneumoniae*, *P. aeruginosa*, *S. epidermidis*, and *S. saprophyticus***	PMMA, glass, and PDMS	Nucleic acid (PCR)	Fluorescence	5 CFU/mL	Affordable, user-friendly, sensitivity	[91]
**AIDS**						
**Anti-HIV-1-antibodies**	PDMS and polystyrene	Immunoassay	Optical	5 pg/mL	Portable, affordable	[92]
**HBsAg, NSP4 and gp41**	PDMS	Immunoassay	Fluorescence	**Sensitivtiy range** HBsAg and gp41: 10^−10^–10^−12^ M NSP4: pM range	Rapid, required small sample	[93]
**p24 protein**	Paper	Immunoassay	Colorimetric	54 fmol	User-friendly, equipment free	[94]
**HIV-RNA**	Membranes, paper and plastic	Nucleic acid (RT-LAMP)	LFIA	2.3 × 10^7^ virus copies/mL	Affordable, user-friendly, sensitive, portable	[95]
**HIV-DNA**	Paper, glass and plastic	Nucleic acid (Isothermal enzymatic amplification)	LFIA	10 copies of HIV DNA	Rapid, sensitive, portable, user-friendly	[96]
**Cardiovascular diseases**						
**CRP**	Silicon	Immunoassay	Fluorescence	1 ng/mL	Sensitive	[97]
**BNP**	Glass and PDMS	Immunoassay	SPR	15 fg	Sensitive, simple	[98]
**CK-MB, cTnI and myoglobin**	Silicon and PDMS	Immunoassay	Chemiluminescence	cTnI: 1.02 pg/mL, CK-MB: 1.37 pg/mL Myo: 4.15 pg/mL	Rapid, sensitive, portable	[99]
**cTnI**	PMMA	Immunoassay	Fluorescence	24 pg/mL	Affordable, portable, sensitive, rapid	[100]
**FABP, cTnI and myoglobin**	Paper	Immunoassay	Fluorescence	FABP: 1.36 ng/mL cTnI: 1.00 ng/mL, Myo: 2.38 ng/mL	Simple, affordable, rapid, robust, portable	[101]
**CRP, NT-proBNP, cTnI and fibrinogen**	PDMS	Aptamers-based assay	Potentiometric	CRP: 0.14 mg/L NT-proBNP: 0.832 pg/mL cTnI: 0.394 pg/mL Fibrinogen: 20.2 mg/dL	Portable, user-friendly, rapid, robust	[102]

## Data Availability

All the revenant data and appropriate references are included in this manuscript itself.
